# Proteomic characteristics and diagnostic potential of exhaled breath particles in patients with COVID-19

**DOI:** 10.1186/s12014-023-09403-2

**Published:** 2023-03-27

**Authors:** Gabriel Hirdman, Embla Bodén, Sven Kjellström, Carl-Johan Fraenkel, Franziska Olm, Oskar Hallgren, Sandra Lindstedt

**Affiliations:** 1 Dept. of Clinical Sciences, Lund University, Lund, Sweden; 2 Wallenberg Center for Molecular Medicine, Lund University, Lund, Sweden; 3 Lund Stem Cell Center, Lund University, Lund, Sweden; 4 BioMS - Swedish National Infrastructure for Biological Mass Spectrometry, Lund University, Lund, Sweden; 5 Department of Infection Control, Region Skåne, Lund, Sweden; 6 Division of Infection Medicine, Department of Clinical Sciences, Lund University, Lund, Sweden; 7 Dept. of Cardiothoracic Surgery and Transplantation, Skåne University Hospital, SE-221 85 Lund, Sweden

**Keywords:** Exhaled breath particles, Proteomics, COVID-19, LC–MS/MS, Breath analysis

## Abstract

**Background:**

SARS-CoV-2 has been shown to predominantly infect the airways and the respiratory tract and too often have an unpredictable and different pathologic pattern compared to other respiratory diseases. Current clinical diagnostical tools in pulmonary medicine expose patients to harmful radiation, are too unspecific or even invasive. Proteomic analysis of exhaled breath particles (EBPs) in contrast, are non-invasive, sample directly from the pathological source and presents as a novel explorative and diagnostical tool.

**Methods:**

Patients with PCR-verified COVID-19 infection (COV-POS, n = 20), and patients with respiratory symptoms but with > 2 negative polymerase chain reaction (PCR) tests (COV-NEG, n = 16) and healthy controls (HCO, n = 12) were prospectively recruited. EBPs were collected using a “particles in exhaled air” (PExA 2.0) device. Particle per exhaled volume (PEV) and size distribution profiles were compared. Proteins were analyzed using liquid chromatography-mass spectrometry. A random forest machine learning classification model was then trained and validated on EBP data achieving an accuracy of 0.92.

**Results:**

Significant increases in PEV and changes in size distribution profiles of EBPs was seen in COV-POS and COV-NEG compared to healthy controls. We achieved a deep proteome profiling of EBP across the three groups with proteins involved in immune activation, acute phase response, cell adhesion, blood coagulation, and known components of the respiratory tract lining fluid, among others. We demonstrated promising results for the use of an integrated EBP biomarker panel together with particle concentration for diagnosis of COVID-19 as well as a robust method for protein identification in EBPs.

**Conclusion:**

Our results demonstrate the promising potential for the use of EBP fingerprints in biomarker discovery and for diagnosing pulmonary diseases, rapidly and non-invasively with minimal patient discomfort.

**Supplementary Information:**

The online version contains supplementary material available at 10.1186/s12014-023-09403-2.

## Introduction

In late December 2019, doctors in Wuhan, China, notified the world of a new cluster of patients with pneumonia of unknown origin [1]. A novel virus, originating from the betacoronavirus family was rapidly sequenced and identified and named severe acute respiratory coronavirus 2 (SARS-CoV-2) causative of the respiratory disease, coronavirus disease 2019 (COVID-19) [2]. The specifics of the pathophysiology of SARS-CoV-2 infection remain poorly understood. Individuals are primarily infected via the airways, where SARS-CoV-2 binds with host angiotensin-converting enzyme 2 (ACE2) via its receptor-binding domain on the spike protein resulting in internalization of the virus into host cells [3]. The subsequent imbalance between the protective and adverse axis of the RAS pathway causes decreased stability of the pulmonary endothelium, inflammatory and thrombotic processes causing respiratory distress [4].

The COVID-19 pandemic highlighted many of the diagnostical challenges of pulmonary disease. Common diagnostical techniques include RT-PCR swabs for viral detection, auscultation, blood work, chest x-ray and computer tomography scans (CT-scans) [5]. However, only bronchoalveolar lavage (BAL) performed during bronchoscopy under sedation can properly detect pathological changes in the otherwise unreachable small airways. Furthermore, all current diagnostical methods have their weaknesses regarding sensitivity, specificity, or potential harm to patients. Novel diagnostical methods in pulmonary medicine are therefore urgently needed.

Exhaled breath is a carrier of valuable information from the respiratory system and analysis of particles and biomarkers provides an attractive such approach. Samples are collected non-invasively and provide a localized sample of the most distal parts of human lungs. Currently two such approaches are actively being researched. Measurements of the volatile compounds in breath, an alcohol breath analyzer being a common example, or the detection and analysis of exhaled breath particles (EBP). Compared with volatile compounds, EBPs can offer more specific insights into disease processes because an array of molecules can be measured. EBPs originate from the respiratory tract lining fluid that covers the epithelial surface of the distal parts of the lung. EBPs are thought to be generated during opening and closing of the distal airways but can also be generated through shear stress [6]**.** The protein composition of EBPs closely resembles that of BAL fluid of which changes in the proteomic composition have been connected to different pulmonary diseases [7]**.**

A few studies have investigated the proteomic characteristics and changes in COVID-19 patients in plasma, BAL, sputum and pulmonary tissue [8–11]. Yet, none have yet investigated the proteomic profile of COVID-19 in EBPs. Furthermore, the proteomic composition of EBPs and alterations in human disease are still poorly understood. We therefore investigated the proteomic composition of EBPs in healthy subjects, in patients with respiratory symptoms but with repeated negative PCR test for COVID-19 infection and in COVID-19 infected patients through high-performance liquid chromatography-mass spectrometry (HPLC–MS/MS) to identify potential biomarkers in exhaled breath for rapid, non-invasive diagnosis and evaluation of pulmonary disease status.

## Methods

### Patients

Patients were recruited prospectively between the 14th of May and 14th of November 2020. A total of 48 patients participated in the study and split into two groups: PCR-verified COVID-19 infection (COV-POS, n = 20), repeat PCR-negative but COVID-19 symptomatic patients (COV-NEG, n = 16) and additionally healthy volunteers were included as controls (HCO, n = 12). Patients were recruited as either inpatients at the infectious disease wards or the emergency department at Skåne university hospital in Sweden. Mean age was 57 years (range 21–70). All patients signed an informed consent form before taking part in the study. The study was approved by the Swedish Ethical Review Authority EPN Dur 2018/129, 2020–018640427 and registered at ClinicalTrials.gov with the trial register number NCT04503057.

### Particle collection

Particles were collected using a PExA 2.0 device (PExA, Gothenburg, Sweden). The instrument uses a two-way valve that allows participants to inhale particle-free air through a HEPA filter and exhale into the instrument. Particles are measured by their size and quantity by an optical particle counter and sized into 16 size bins and collected on a membrane by an inertial impactor within the device. The bin sizes averages ranges from 0.33 µm to 3.67 µm. Exhaled flow and volume are measured by an ultrasonic flow meter. A breathing maneuver, previously described, was used for the EBP collection until a goal amount of 120 ng of sampled particles had been collected [6, 12]. The particles are measured and expressed as number of particles per volume (PEV) and relative counts per particle size. All samples were immediately transferred after collection and stored at − 80 °C for later analysis. No participants reported any adverse events in connection to EBP sampling.

### Statistical analysis of particle data

All statistical test related with PEV were done using Graphpad Prism 9 (Graphpad Software, San Diego, CA). Descriptive statistics in the form of median and interquartile range was used for particle and patient data. Kruskal–Wallis test with Dunn’s post hoc test was used to compare PEV between groups. For statistical analysis between correlation of PEV to age the data were first transformed into its natural logarithms and then analyzed using Pearson parametric correlation coefficients and reported as R2. For comparison of PEV between sexes Mann–Whitney-U was used. For comparison of relative particle sizes between groups log transformed particle data was analyzed with a mixed effects model REML and Tukey’s multiple comparisons test. Statistical significance was defined as ****p < 0.0001, ***p < 0.001, **p < 0.01, *p < 0.05 and NS (p > 0.05).

### Sample preparation for LC–MS/MS

EBP samples were incubated in 2% sodium dodecyl sulfate (SDS, Sigma-Aldrich, St. Louis, USA) in 50 mM Triethylammonium bicarbonate (TEAB, Thermo Fisher Scientific) at 37 °C for 2 h with subsequent addition of 400 mM dithiothreitol (Sigma-Aldrich) and further incubation for 45 min.. Alkylation was performed in the dark for 30 min with the addition of 800 mM iodacetamide (Sigma-Aldrich) after which 12% aqueous phosphoric acid was added to a final concentration of 1.2%. Proteins were collected onto S-TRAP columns (Protifi, Farmingdale, USA) with a mixture of 90% methanol and 100 mM TEAB. Digestion of proteins was performed with 1 µg of Lys-C (Lys-C, Mass Spec Grade, Promega, Fitchburg, USA) incubated at 37 °C for 2 h after which 1 µg of trypsin (Promega sequence grade) was added overnight with addition of 0.45 µg Trypsin after 12 h. Peptides were then eluted with 50 mM TEAB, 0.2% formic acid (FA, Sigma-Aldrich) and 50% acetonitrile (ACN, Sigma-Aldrich) with 0.2% formic acid and dried by speedvac (Eppendorf, Hamburg, Germany) at 45 °C and re-dissolved in 20 uL of 0.1% FA and 2% ACN solution.

### LC–MS/MS

Digested peptides were separated with nanoflow reversed-phase chromatography with an Evosep One liquid chromatography (LC) system (Evosep One, Odense, Denmark) after loading the samples on Evosep tips. Separation was performed with the 60 SPD method (gradient length 21 min) using an 8 cm × 150 µm Evosep column packed with 1.5 μm ReproSil-Pur C18-AQ particles. The Evosep One was coupled to a captive source mounted on a timsTOF Pro mass spectrometer from Bruker Daltonics (Billerica, Massachusetts, USA). The instrument was operated in the DDA PASEF mode with 10 PASEF scans per acquisition cycle and accumulation and ramp times of 100 ms each. Singly charged precursors were excluded, the ‘target value’ was set to 20,000 and dynamic exclusion was activated and set to 0.4 min. The quadrupole isolation width was set to 2 Th for m/z < 700 and 3 Th for m/z > 800.

### LC–MS/MS data analysis

MaxQuant (v2.0.20, Max Planck institute of biochemistry, Munich, Germany) using the Andromeda database search algorithm was used to analyze raw MS data [13]. Spectra files were searched against the UniProt filtered and reviewed human protein database using the following parameters: Type: TIMS-DDA LFQ, Variable modifications: Oxidation (M), Acetyl (Protein N-term) and Fixed modifications: Carbamidomethyl (C). Digestion, Trypsin/P, Match between runs: False. FDR was set at 1% for both protein and peptide levels. MS1 match tolerance was set as 20 ppm for the first search and 40 ppm for the main search. Missed cleavages allowed was set to 2. Subsequently the Spectra files were searched against the UniProt SARS-CoV-2 proteome database (Proteome ID: UP000464024) using the same parameters. Data was first normalized with NormalyzerDE using robust linear regression normalization [14]. Perseus (v2.0.5.0, Max Planck institute of biochemistry, Germany) and RStudio (v4.2.0, RStudio, Boston, MA, US) were used for downstream analysis of proteomics data. Proteins denoted as decoy hits, contaminants, only identified by site were removed. Next proteins identified in less than 45% of samples in at least one group were removed. Significant differences in protein intensities between groups were determined with an ANOVA q-value of < 0.05 and post hoc Tukey’s test of the log2-transformed LFQ intensities. Differentially expressed proteins were determined using and s0 of 0.1 and FDR of 0.05. For the heatmap LFQ values were normalized with a Z-score and rendered in RStudio using the pheatmap package using euclidean clustering. Protein–protein interaction and Reactome Pathways were analyzed using STRING v11.5 using the stringApp within Cytoscape v3.9.1. Subcellular location determined with CellWhere v.1.1 [15]. Statistical significance was defined as ****p < 0.0001, ***p < 0.001, **p < 0.01, *p < 0.05 and NS (p > 0.05).

### Machine learning classification model

A diagnostic classification model was built using the R CARET package (version 6.0–93). For the machine learning analysis, missing values were first imputed in Perseus with a width of 0.3 and a down shift of 1.3. Independent feature selection was used within Perseus and based on ANOVA scores and least number of missing values. The top 11 proteins as well as each subject´s PEV count was determined to give the smallest error percentage. The following biomarker panel was selected: ORM1, IGHG1, CAPN1, CASP14, PEV, IGLC6, APOA1, TF, IGKC, EPPK1, SFTPB and IGHA1 and the data subsequently exported into R. The cohort was split randomly in a 60/40 split for training (n = 22) and testing (n = 12) respectively with subjects classified as either positive (COV-POS, n = 12) or negative (COV-NEG and HCO, n = 22). A random forest model was trained on the training set with tenfold cross validation repeated 100 times and using 1000 trees. Receiver operating characteristic (ROC) was used to select the optimal number of randomly drawn candidate variables (mtry) and set at 2. The results of the model are based on application of the model on the test set and reported as accuracy, sensitivity and specificity and area under the ROC curve (AUC-ROC).

## Results

### Patient demographics

Median age and sex were similar between COV-POS and COV-NEG with median age being lower in HCO. COV-POS patients had a higher incidence of obesity and asthma in comparison to COV-NEG and HCO. Symptomatology were similar between COV-POS and COV-NEG regarding fever, throat pain, stomach pain and myalgia but differed significantly regarding dyspnea with 95% of COV-POS patients reporting it as a symptom. No symptoms were reported in the HCO group. EBP measurements were on average sampled on day 7 post COVID-19 positive test but ranged between 1 and 9 days. A summary of participant information can be found in Table 1.

**Table 1:** Patient characteristics

Characteristics	COV-POS	COV-NEG	HCO
Number of participants	20	16	12
Sex: Male	10 (50%)	8 (50%)	4 (44%)
Age (Median)	56 (IQR: 53–64)	69 (IQR: (53–80)	44 (IQR: 29–46)
Days since symptom debut*	8 (IQR: 3.75–10)	2 (IQR: 1–4.75)	0
Clinical diagnosis			
Infectious etiology			
Viral	20 (100%)	5 (31.3%)	0 (0%)
Bacterial	0 (0%)	4 (25%)	0 (0%)
Unknown	0 (0%)	5 (31,3%)	0 (0%)
Non-infectious respiratory symptoms	0 (0%)	2 (12.5%)	0 (0%)
Comorbidities			
Asthma	1 (5%)	0 (0%)	0 (0%)
COPD	3 (15%)	1 (6.25%)	0 (0%)
Obesity	10 (50%)	4 (25%)	1 (8.3%)
Symptoms			
Coughing	14 (70%)	8 (50%)	0 (0%)
Fever	11 (55%)	6 (38%)	0 (0%)
Throat pain	2 (10%)	3 (19%)	0 (0%)
Stomach pain	4 (20%)	4 (25%)	0 (0%)
Dyspnea	19 (95%)	9 (56%)	0 (0%)
Myalgia	3 (15%)	1 (6%)	0 (0%)
Hospitalized	20 (100%)	7 (44%)	0 (0%)

Characteristics for patients with PCR-verified COVID-19 infection (COV-POS), COVID-19 PCR-negative patients with respiratory symptoms (COV-NEG) and healthy controls (HCO)

*IQR* Interquartile rang**e**

*Or days since seeking medical care if unknown. Descriptive statistics presented as number of patients and percentage

### Analysis of exhaled particle data

**Fig. 1:**
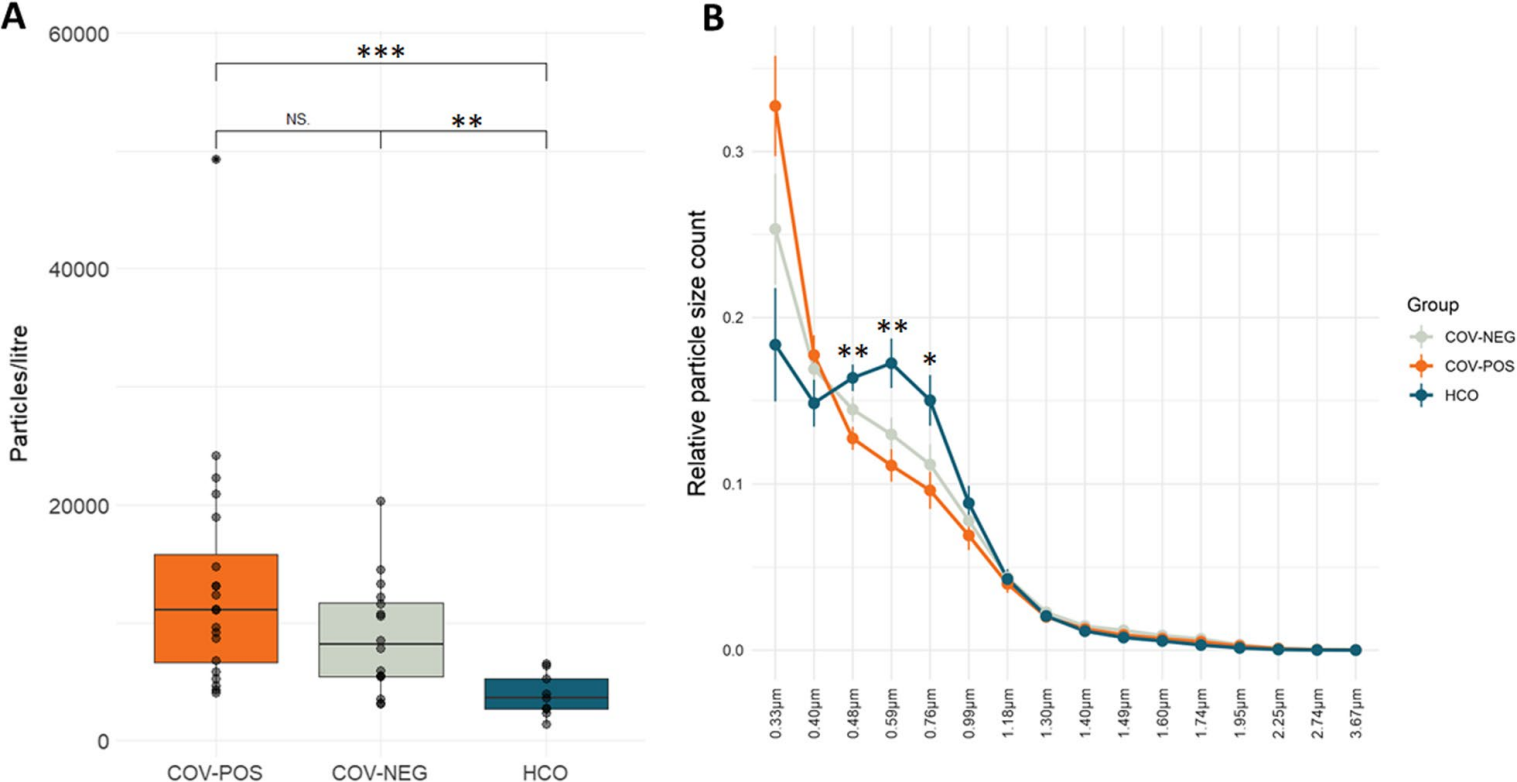
Exhaled breath particle concentrations and particle size distributions differed significantly between symptomatic and healthy patients. Particles in exhaled air were measured using an optical particle counter. **A** Particles per exhaled volumes (PEV) for patients with PCR-verified COVID-19 infection (COV-POS), patients with respiratory symptoms but with > 2 negative polymerase chain reaction (PCR) tests for COVID-19 (COV-NEG) and healthy controls (HCO) Data shown as individual values (black dots) with lower and upper boundary of boxplots representing 25th and 75th percentile. Statistical significance was tested with Kruskal–Wallis test with Dunn’s multiple hypothesis testing correction. **B** Relative particle size counts per particle size bin for COV-POS, COV-NEG and HCO. Data are shown as mean ± standard error of mean. Statistical significance was tested using ANOVA with Tukey’s multiple comparisons correction and significance values are shown between COV-POS and HCO. Statistical significance was defined as ****p < 0.0001, ***p < 0.001, **p < 0.01, *p < 0.05 and NS (p > 0.05)

EBPs were collected and particles per exhaled volume (PEV) were measured over time, summed, and compared between groups. There was a significant increase in PEV in COV-POS and COV-NEG patients compared to HCO. COV-POS exhaled a median of 11,902 particles (Interquartile range (IQR): 6119–17,893) and COV-NEG a median of 8,159 (IQR: 5406–12,000) compared to a median of 3,622 (IQR: 2506–5790) in the HCO group. Figure 1A demonstrates this large intra-group variation in IQR range in PEV in COV-POS and COV-NEG. Furthermore, there was no correlation between PEV and age (r^2^ = 0.06954) or between sexes in PEV (p = 0.3254).

Patients with respiratory symptoms (COV-POS and COV-NEG) skewed towards exhaling smaller particles in comparison to the HCO group. In these patients, particle bin size 1, accounting for particles with a median diameter of 0.33 µm constituted on average 33% of total exhaled particles compared to just 18% for the same particle bin size in HCO. The HCO group presented with a bimodal distribution of relative particle size distribution in comparison with the right skewed distribution in the symptomatic groups. Figure 1B presents particle size distributions between the three groups.

### LC–MS/MS based protein identification of exhaled particles

Patient samples with 100 ng or more collected particles were selected for LC–MS/MS protein identification yielding a total of 34 samples for further analysis. 12 samples each from the COV-POS and COV-NEG groups were analyzed and 10 samples from the HCO group. A flow chart summarizing sample exclusion can be seen in Additional file 1: Fig. S1. In total 267 unique proteins could be identified across all three groups after exclusion of potential contaminants. 146 proteins were present in 45% of samples in at least one group, identifying immunoglobulin heavy constant gamma 3 (IGHG33) as the only unique protein found in the COV-POS group, identified in 50% (n = 6) of all samples in the group. Mean number of proteins identified per sample was 110.1 (SD: 15.8). No viral SARS-CoV-2 proteins could reliably be detected in any of the samples.

### LC–MS/MS quantitative proteomics of exhaled particles

**Fig. 2:**
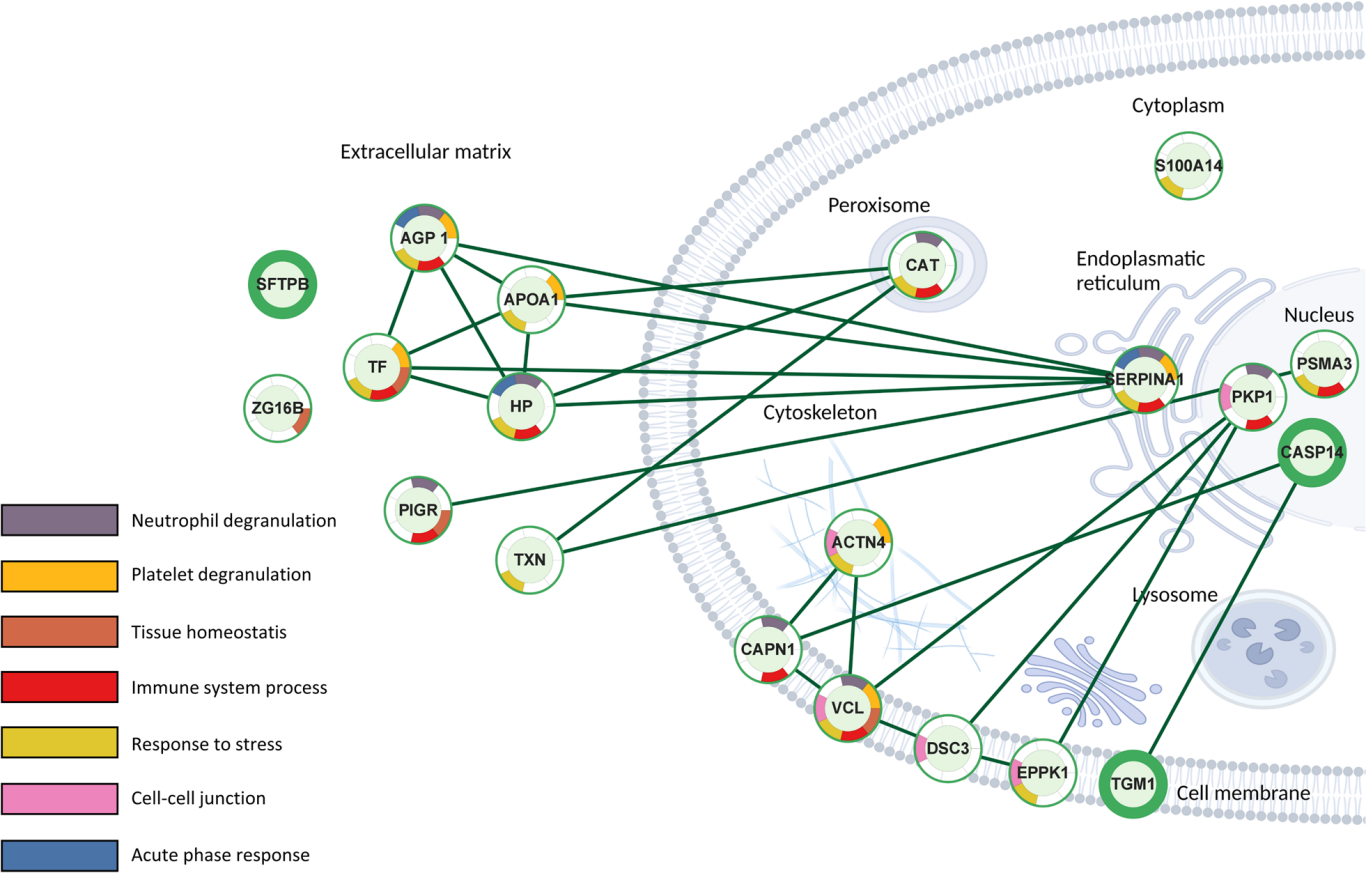
Schematic of protein–protein interaction network with subcellular location and Reactome Pathways for significantly differentiated proteins. Protein–protein interaction and Reactome Pathways created with STRING v11.5 inside Cytoscape v3.9.1 and subcellular location determined with CellWhere v1.1. Only significantly differentiated proteins found within the STRING database are mapped. Image created with biorender

**Fig. 3:**
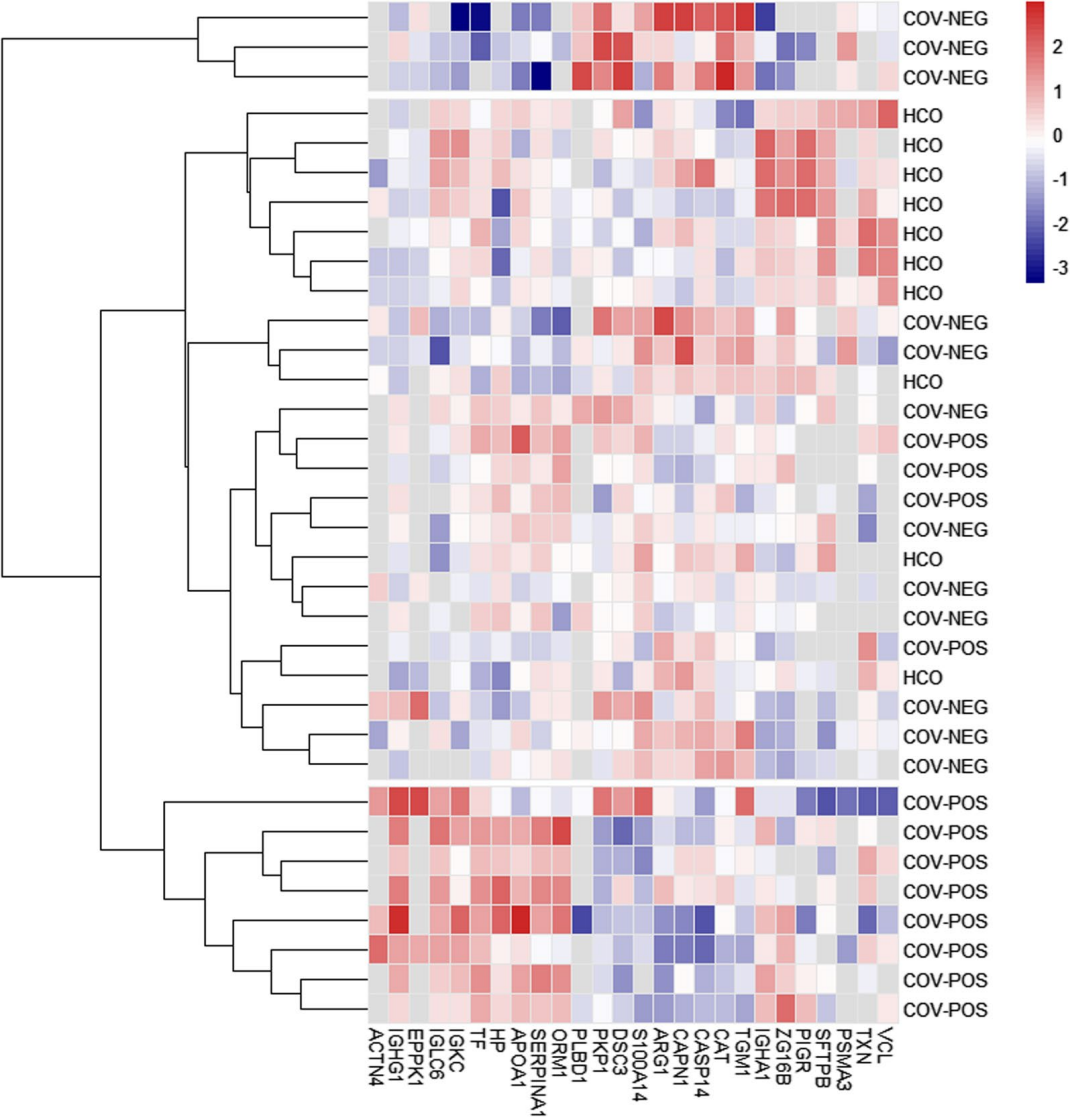
COVID-19 positive patients exhibited a clustered expression profile of exhaled breath proteins. Protein intensities of the 27 differentially expressed proteins were log10 transformed, normalized with a Z-score and displayed as colors ranging from blue to red with white boxes indicating missing values. Rows are clustered using Euclidean distance and cluster into three distinct expression profiles indicated by gap between rows. Samples are grouped into patients with PCR-verified COVID-19 infection (COV-POS), patients with respiratory symptoms but with > 2 negative polymerase chain reaction (PCR) tests for COVID-19 (COV-NEG) and healthy controls (HCO)

**Fig. 4:**
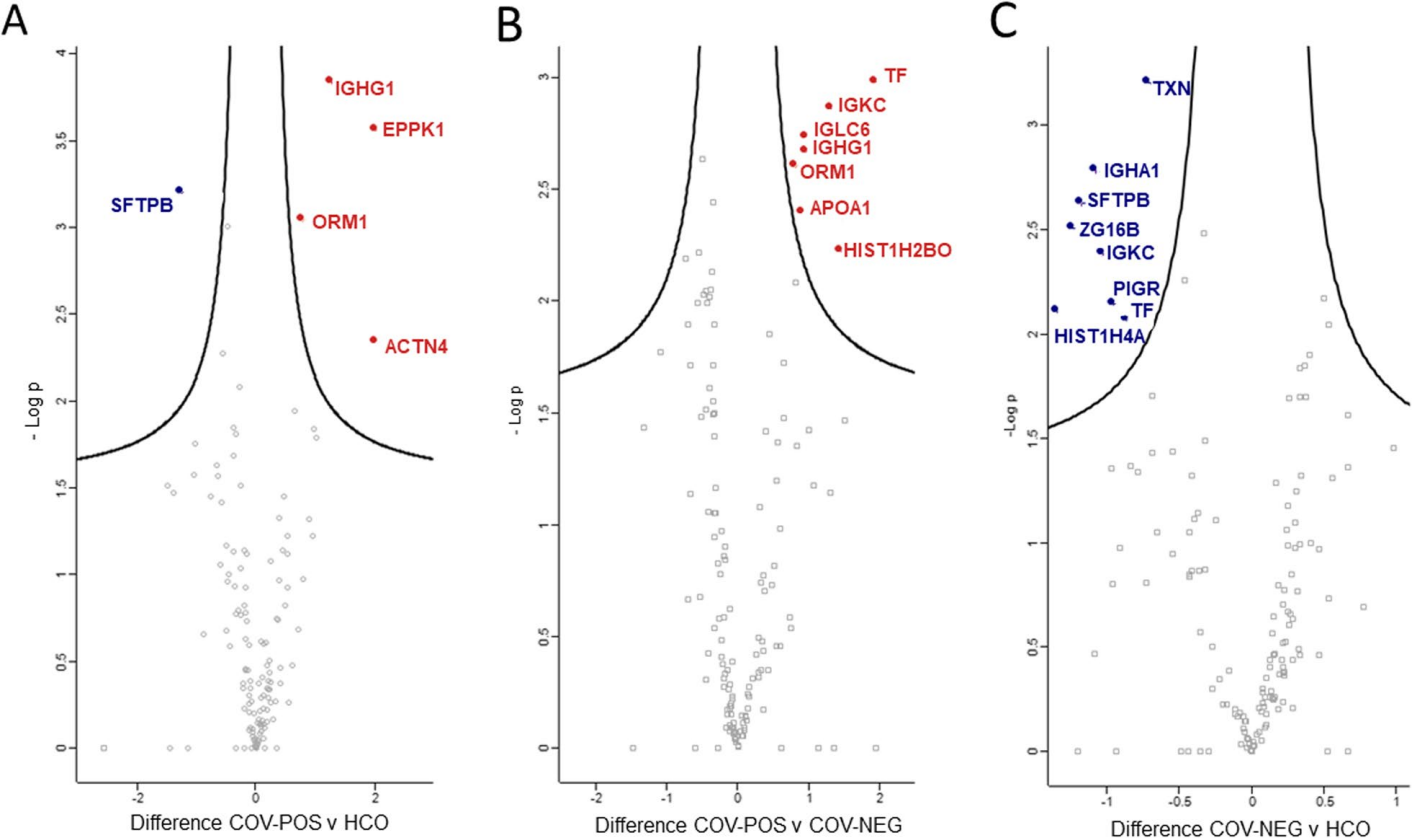
COVID-19 positive patients showed statistically significant differentially expressed proteins in exhaled breath. X-axis show difference in intensities and y-axis negative log p-value calculated using a student’s *t*-test. Significantly differentially expressed upregulated proteins are highlighted in red and downregulated proteins are highlighted in blue. **A** Volcano plot of differentially expressed proteins between PCR-verified COVID-19 infection (COV-POS) and healthy controls (HCO). **B** Volcano plot of differentially expressed proteins between COV-POS and patients with respiratory symptoms but with > 2 negative polymerase chain reaction (PCR) tests for COVID-19 (COV-NEG). **C** Volcano plot of differentially expressed proteins between patients with respiratory symptoms but with > 2 negative polymerase chain reaction (PCR) tests for COVID-19 (COV-NEG) and healthy controls (HCO)

**Fig. 5:**
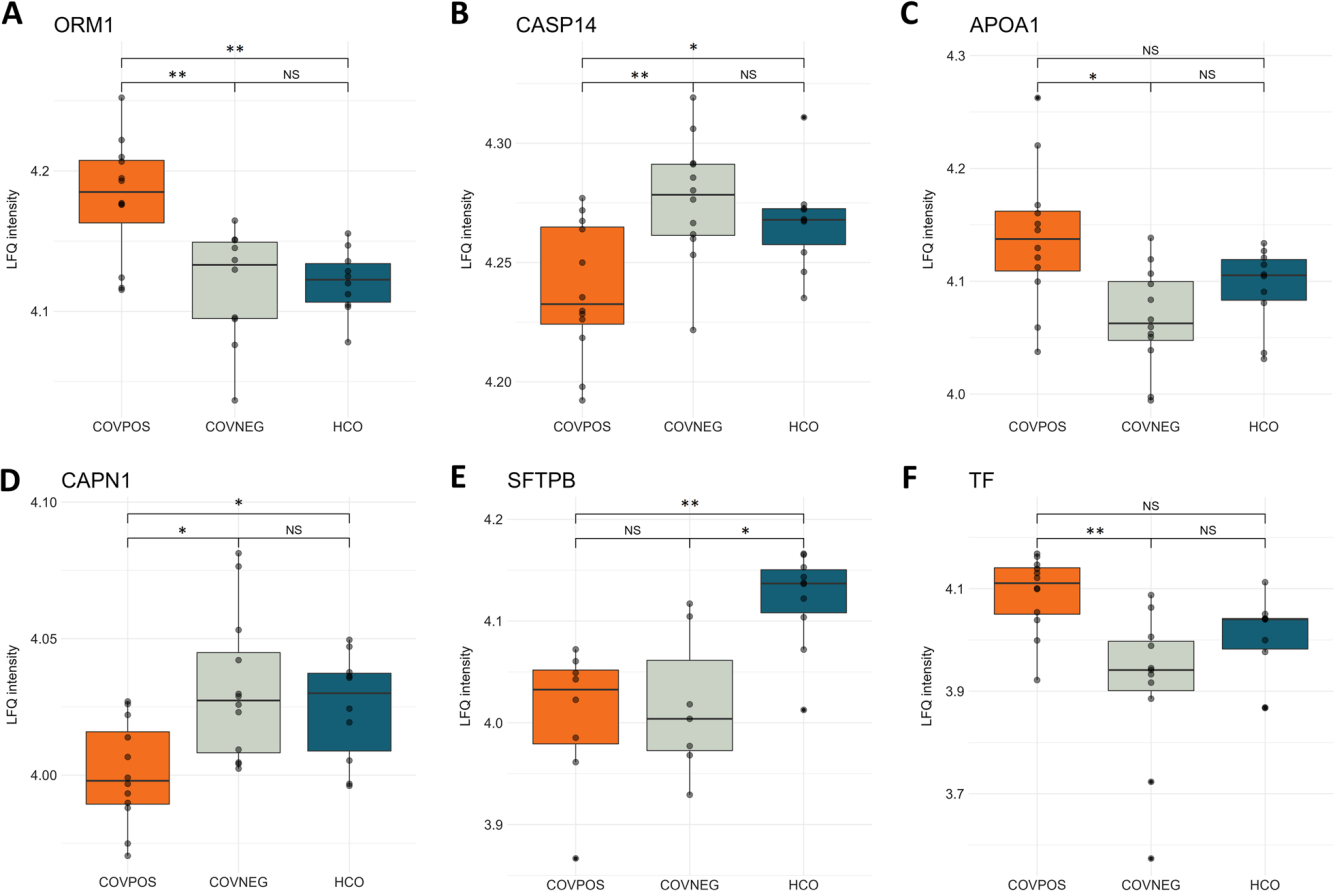
The six most abundant differentially expressed proteins between groups. Differences in protein expression between PCR-verified COVID-19 infection (COV-POS), patients with respiratory symptoms but with > 2 negative polymerase chain reaction (PCR) tests for COVID-19 (COV-NEG) and healthy controls (HCO). Boxplots of COV-POS (orange), COV-NEG (grey) and HCO (blue) for **A** Alpha-1-acid glycoprotein 1 (ORM1), **B** Caspase-14 (CASP14), **C** Apolipoprotein 1 (APOA1), **D** Calpain 1 (CAPN1), **E** Pulmonary surfactant associated protein B (SFTPB), and **F** Transferrin (TF). Data are presented as individual values (black dots). Line in boxplots represents mean and the lower and upper boundary of boxplots representing 25th and 75th percentile with whiskers below and above boxes representing 10th and 90th percentile, respectively. Statistical significance was tested with ANOVA and Tukey’s honest significance test and defined as ****p < 0.0001, ***p < 0.001, **p < 0.01, *p < 0.05 and NS (p > 0.05)

Subsequently, identified proteins were quantified with label free quantification (LFQ) of exhaled particles. In total 26 proteins were identified as significantly differentially expressed and summarized in Table 2. Significantly differentiated proteins were mainly extracellular proteins, as shown in Fig. 2, but included proteins localized to the cell membrane and intracellular proteins. Reactome pathway analysis revealed differentially expressed proteins related to, among other things, the innate immune system as well as neutrophil and platelet degranulation. Clustering analysis of significantly differentiated proteins among groups revealed three distinct groups, of which 67% (n = 8) of COV-POS patients compromised one cluster as shown in Fig. 3. A second cluster was comprised of three COV-NEG samples and the last cluster of the remaining samples, including four COV-POS samples. Nine proteins were significantly upregulated in COV-POS patients in comparison to the COV-NEG and HCO groups and are shown in Fig. 4A, B. In comparing COV-NEG to HCO, eight proteins were found to be significantly downregulated as shown in Fig. 4C. The upregulated proteins included three immunoglobulins: Immunoglobin kappa constant (IGKC), Immunoglobulin heavy constant gamma 1 (IGHG1) and immunoglobin lambda constant 3 (IGLC3) as well as Epiplakin (EPPK1), a protein involved in wound healing. Figure 5 presents boxplots of proteins significantly differentially expressed of particular interest in COV-POS patients and include Serotransferrin (TF, F), Apolipoprotein A-I (APOA1, C), Caspase-14 (CASP14, B), Calpain-1 (CAPN1, D), and Alpha-1-acid glycoprotein 1 (ORM1 1, A), a modulator of the immune system during the acute-phase reaction. Pulmonary surfactant-associated protein B (SFTPB, E) was significantly downregulated in COV-POS and COV-NEG patients versus the HCO group.

**Table 2:** Significantly differentially expressed proteins

Gene names	Protein names	ANOVA	q-value	Mean difference		Andromeda score
				COV-POS	COV-NEG	HCO
IGHG1	Ig gamma-1 chain C region	0.004	4.4	-3.0	-4.4	323
IGKC	Ig kappa chain C region	0.011	3.1	-3.1	2.2	323
ORM1	Alpha-1-acid glycoprotein 1	0.012	2.8	− 2.8	− 2.6	165
SFTPB	Pulmonary surfactant-associated protein B	0.016	− 2.7	− 2.2	2.7	69
TF	Serotransferrin	0.021	2.9	− 2.9	0.0	323
IGHA1	Ig alpha-1 chain C region	0.022	1.3	− 2.9	2.9	323
CASP14	Caspase-14	0.027	− 2.6	2.6	1.5	323
EPPK1	Epiplakin	0.029	2.6	− 1.4	− 2.6	292
CAPN1	Calpain-1 catalytic subunit	0.033	− 2.3	2.3	1.4	61
IGLC6	Ig lambda-6 chain C region	0.034	2.5	− 2.5	1.4	229
APOA1	Apolipoprotein A-I	0.036	2.4	− 2.4	0.0	308
CAT	Catalase	0.036	− 1.4	2.2	− 2.2	323
DSC3	Desmocollin-3	0.037	− 2.2	2.2	− 1.7	270
VCL	Vinculin	0.040	− 1.8	− 1.8	1.8	52
PKP1	Plakophilin-1	0.041	− 2.1	2.1	0.0	323
TGM1	Protein-glutamine gamma-glutamyltransferase K	0.043	− 1.8	1.8	− 1.5	261
PSMA3	Proteasome subunit alpha type-3	0.043	− 2.1	2.1	1.7	88
ZG16B	Zymogen granule protein 16 homolog B	0.043	0.0	− 2.2	2.2	323
ARG1	Arginase-1	0.044	− 2.1	2.1	0.0	323
SERPINA1	Alpha-1-antitrypsin	0.044	2.2	− 2.2	0.0	323
ACTN4	Alpha-actinin-4	0.046	2.0	− 1.3	− 2.0	65
S100A14	Protein S100-A14	0.047	− 1.9	1.9	0.0	227
TXN	Thioredoxin	0.048	− 1.3	− 1.8	1.8	84
PIGR	Polymeric immunoglobulin receptor	0.048	− 1.6	− 1.6	1.6	109
HP	Haptoglobin	0.049	2.0	0.0	− 2.0	188
PLBD1	Phospholipase B-like 1	0.049	− 1.9	1.9	0.0	76

Summary of significantly differentially expressed proteins between PCR-verified COVID-19 infection (COV-POS), COVID-19 PCR-negative patients with respiratory symptoms (COV-NEG) and healthy controls (HCO) and their adjusted p-value (ANOVA q-value) and Andromeda score from the MaxQuant search engine

### Machine learning classification of samples

**Fig. 6:**
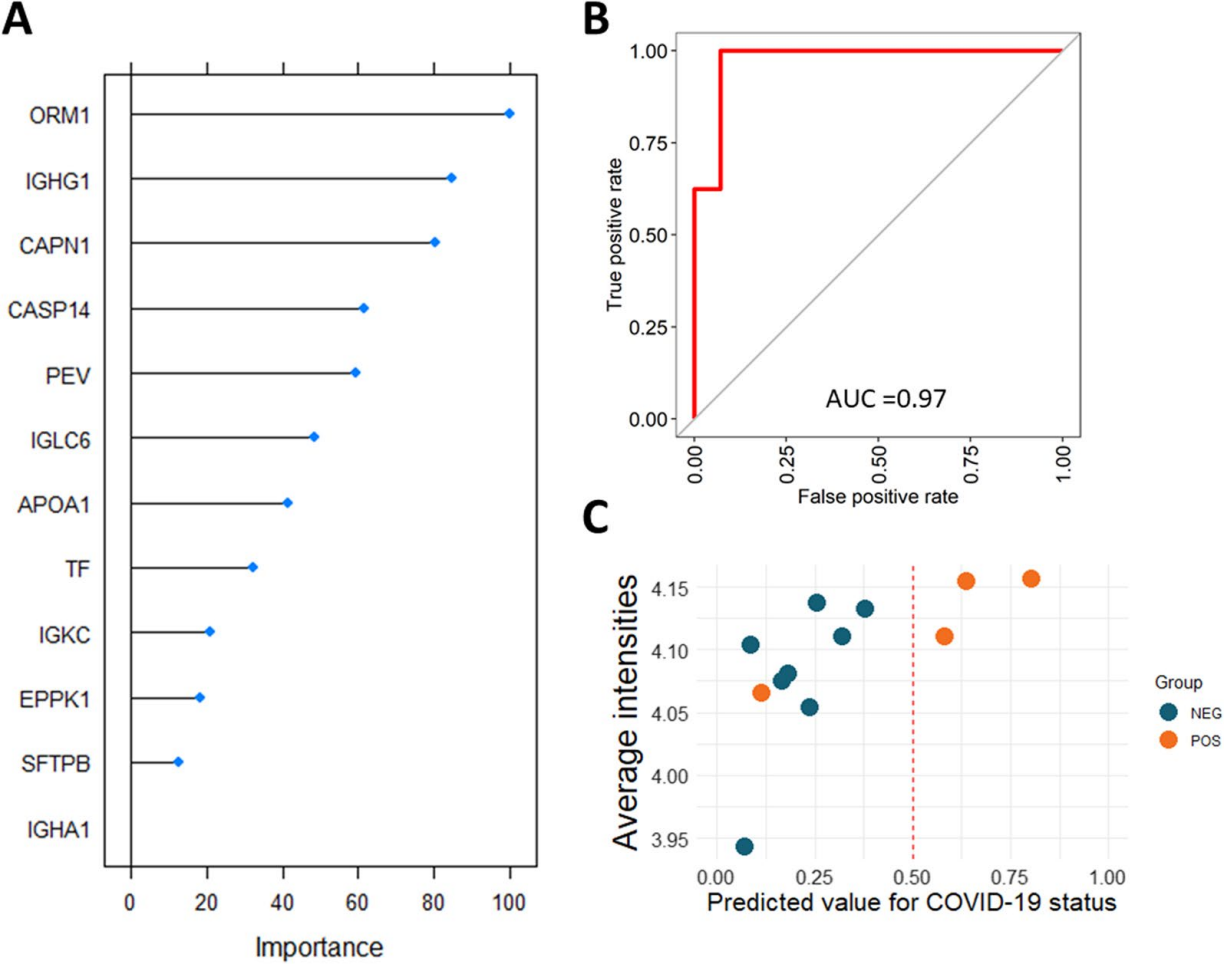
Random forest machine learning model classification of EBP data to predict COVID-19 disease status. **A** Scaled variable importance for the classification model ranked by mean decrease in accuracy of the model. **B** Receiver operating characteristics of the random forest model in the training cohort. **C** Outcome of the model on the test cohort shown as predicted value for COVID-19 status with 1.0 as certain and < 0.5 as negative for COVID-19. Only one sample was misclassified by the model

A machine learning (ML) random forest classification model was built using 11 proteins found in all groups and subjects PEV counts. For training, 22 samples were randomly selected, and variables ranked by the ML model according to importance (Fig. 6A). The ROC-AUC for the training data was determined to be 0.97 (CI 0.88–1.06). Next the model was tested on the remaining 12 samples and achieved an accuracy of 0.92 (CI 0.62–0.99), with only one COVID-19 positive sample misclassified as negative, in the testing cohort. The misclassified sample belonged to a 51-year-old female that had tested positive 8-days prior to particle collection and was subsequently discharged from the hospital the following day, possibly affecting the classification. Sensitivity for the model was determined as 75% and specificity as 100%. AUC-ROC in the training data was 0.97 (CI 0.88–1.06) and AUC-ROC of the test data 0.81 (CI 0.52–1.1).

## Discussion

This study presents a novel method for analyzing the proteome of exhaled breath particles for diagnosis and characterization of disease. Sampling of approximately 100 ng of exhaled particles allowed for detection of an average of 110 proteins per sample. This is in stark comparison to the commonly used exhaled breath condensate (EBC) analysis, where the low protein concentrations often require pooling of samples to identify similar numbers of proteins [16, 17]. We achieved a deep proteomic profiling of EBP across the three groups with proteins involved in immune activation, acute phase response, cell adhesion, blood coagulation, and known components of the respiratory tract lining fluid (RTLF), among others. EBP sampling moreover allowed for the analysis of the respiratory tract health status in two-dimensions. Both in terms of the proteome of the exhaled particles as well as the particle concentrations and size distributions, which in turn have previously been implicated in respiratory disease [18].

In accordance with other published work, we identified an increase in particle production in patients with respiratory symptoms [18, 22, 23]. Particle production is thought to depend on the bulk rheological properties of RTLF. Studies have shown that modifications to the viscoelastic properties of RTLF, such as inhalation of isotonic saline, significantly change particle production, possibly explaining the increases in particle production found in our study [24]. COVID-19 patients exhibited a significant increase in particle production with a tendency towards the smaller particles. Similarly, COV-NEG patients, meaning patients with respiratory symptoms, likewise presented with a slightly lower increase in particle concentrations suggestive of a disease-dependent variation in surfactant composition. Thus, EBP collection is a promising new method for monitoring pulmonary health status over the course of an infection and has previously been investigated in other diseases [25, 26].

Proteins in the RTLF originate from various sources, including respiratory epithelial cells, resident inflammatory cells, and plasma proteins that leak from the capillary membrane. Proteins in the RTLF have broad mechanistic roles, including microbial defence, wound healing, maintaining the viscoelastic properties of the fluid, and nutrient transport, among others. Understanding and being able to monitor the proteomic changes would therefore be an attractive approach for diagnosis and disease monitoring directly from the infection or pathological focus. Proteomic analysis of BALF is one such approach and allows direct sampling of the RTLF, yet it is highly invasive and can only be performed on a limited scale in the clinic and for biomarker research. Previously reported overexpressed proteins in BALF in COVID-19 patients, correspond well to our findings, particularly for the six most abundant proteins in all samples [27]. Of particular interest in biomarker research for infectious diseases are acute phase proteins, which increase in expression in response to inflammation. Three acute phase proteins were significantly overexpressed in EBP in COVID-19 patients compared to COV-NEG and HCO. These proteins were ORM1, alpha 1 antitrypsin, and haptoglobin. Of these three, ORM1 was identified in almost all samples and significantly increased in the COV-POS group compared with both COV-NEG and HCO in EBP. ORM1 is mainly excreted from hepatic cells in response to various stress-related stimuli, but extrahepatic production has been reported, such as from alveolar type II cells upon lipopolysaccharide (LPS) induction in rats [28]. ORM1 has previously been of interest for pulmonary infections. Hamid et al. found that ORM1 plasma levels were a sensitive and specific biomarker for mortality prediction in children with pneumonia [29]. Plasma proteomic studies in COVID-19 patients, have similarly found increased expression levels, and correlations to disease severity have been reported [27]. Sampling of ORM1 from the RTLF using EPB collection, therefore, presents an opportunity for direct detection of stress-related changes in the lungs, possibly long before such changes can be seen in plasma or detected through physiological changes (see Additional file 2).

Of further interest in biomarker discovery in COVID-19 are stress response proteins. APOA1 is such a marker and was found to be significantly increased between COV-POS and COV-NEG. It has previously been implicated in the inflammatory response and immune regulation, including antioxidative and antiviral properties and is expressed in the lung epithelium [30–33]. Recently published plasma proteomic studies of COVID-19, in contrast, report finding decreased levels of APOA1 [9, 34]. However, in BAL, increases in concentrations have been reported correlating with lymphocyte concentrations or severity of lung injury [35, 36]. APOA1 might therefore be a highly specific diagnostic protein for lung injury with upregulation localized to the RTLF and, together with ORM1 forms a signature of an early response to pulmonary infection. Other stress response proteins include serotransferrin (TF). It is an iron-binding transported glycoprotein mainly synthesized by hepatocytes and, to a certain degree, in lymphocytes [37, 38]. In the human lung, TF is primarily synthesized and excreted by pulmonary epithelial cells and submucosal glands, and alveolar macrophages [39]. TF in BAL have been reported to be present in much higher concentrations in comparison with plasma, making it a particularly interesting protein in EBP research [40]. TF is mainly known for the iron-binding activity. However, new evidence points to its activity within the coagulation cascade, interfering with antithrombin/SERPINC1 and factor XIIa leading to increased coagulation indicating an increased tendency for procoagulant disorders in COVID-19 patients [41]. Increased levels of TF have been reported in BAL fluid in patients with ARDS and patients at risk of ARDS while simultaneously being downregulated in plasma, presenting it as an exciting biomarker candidate in EBP [42]. Furthermore, TF abundance was discordantly downregulated in COV-NEG patients in comparison to HCO, suggestive of a COVID-19 causative specific increase in EBP.

COVID-19 utilizes ACE2 receptors to access and infect pulmonary surfactant-producing alveolar type II (ATII) cells [43]. Subsequent viral-induced lysis and apoptosis of ATII cells and consequent loss of surfactant in COVID-19 patients are an important part of the pathology and are linked to diffuse alveolar damage, protein leakage and hyaline membrane formation [44]. In accordance, levels of SFTPB were significantly decreased in the EBP of diseased lungs, indicating that EBP collection and analysis could offer a simple and effective way of sampling the health status of the distal parts of the lungs, which has not been possible in the clinic before. Reduction of SFTPB levels in the alveolar space has been shown to precede the clinical development of ARDS and decrease the surface tension, perhaps an important mechanism for increased particle production in these individuals [45, 46]. Surfactant is mainly composed of Dipalmitoylphosphatidylcholine and has previously been studied in EBP, showing decreases in smokers' lungs [47]. Exogenous administrated surfactant has been shown to improve oxygenation in COVID-19 ARDS, and early administration could provide a benefit, showing the potential for EBP collection and analysis in rapidly aiding clinicians in driving therapeutic decisions. [48].

No viral proteins were identified in any of the samples by LC–MS/MS analysis. Previous attempts at detecting viral SARS-CoV-2 proteins using the more sensitive PCR analysis corroborate these results with detection of SARS-CoV-2 in only 3 of 25 samples using the standardize breathing maneuver [19]. Although attempts at identifying SARS-CoV-2 proteins by LC–MS/MS methods have been successful, for example in gargle solution and nasopharyngeal nose swaps, these represent samples from the upper respiratory tract, which may explain the lack of detection in the lower tract sampling method of EBP [20, 21].

In order to examine the diagnostic potential of EBP for lung diseases we composed an integrated proteomic biomarker panel with particle production counts for a machine learning algorithm. The classifier consequentially achieved an overall accuracy of 92% in our test data illustrating the robust potential for future protein and particle production fingerprints in diagnosing pulmonary disease, rapidly and non-invasively with minimal patient discomfort.

While this study shows promising results for the use of EBP it includes a few limitations. Firstly, the study includes a relatively small sample size. Correct sensitivity and specificity values for the machine classifier are therefore difficult to accurately quantify and more differences in EBP expression could be undetected due to low power. Furthermore, days since symptom onset were unmatched between groups, possibly affecting PCR readout accuracy of COVID-19 and proteomic changes in EBP. All patients with negative COVID-19 PCR tests have therefore been reviewed for the presence of a positive COVID-19 tests in the days during the patients entire hospital stay in the days following EBP sampling. Future studies of EBP in COVID-19 and similar diseases will be needed to improve and further evaluate the diagnostical accuracy.

EBP collection allows for the detection of upregulated proteins localized to the lung milieu and enables clinicians to obtain direct insight into disease-related activity at the source. Our data show promising results to stratify protein expression patterns to distinguishing healthy RTLF from diseased. Together with particle production data, a complete picture of RTLF composition and viscoelastic function can be discerned and used to drive clinical decision-making.

## Conclusion

Mass-spectrometry-based proteomic analysis of exhaled breath particles enables exciting new possibilities for pulmonary diagnostics and biomarker discovery. Particle production is indicative of pulmonary disease status, and protein composition differs significantly between healthy and infected patients. Potential biomarkers in EBP include extracellular acute-phase proteins, decreases in surfactant-associated proteins, and intracellular proteins. Furthermore, we have shown promising potential for the use of an EBP biomarker panel together with particle concentration for diagnosis of COVID-19 as well as a robust method for protein identification in EBP.

## Supplementary Information

**Additional file 1: Figure S1.** Flow chart of patient inclusion and sample exclusion. In total 48 subjects were recruited and split into three groups based on symptoms and COVID-19 PCR test results. Subsequently 13 samples were excluded due to insufficient particle collection (< 100 ng of sampled material). One sample in the Healthy control group further failed the mass spectrometry analysis due to technical reasons. The remaining samples where then used for training and testing a machine learning classifier.

**Additional file 2: Table S1. **LC-MS/MS identified proteins with their statistical differences. Summary of all comparisons between PCR-verified COVID-19 infection (COV-POS), PCR-negative patients with respiratory symptoms (COV-NEG) and healthy controls (HCO) and their adjusted p-value (ANOVA q-value) and Andromeda score from the MaxQuant search engine.

## Data Availability

The datasets supporting the conclusions of this article are available at the ProteomeXchange Consortium via the PRIDE partner repository with the dataset identifier PXD039058.

## Abbreviations

SARS-CoV-2: Severe acute respiratory syndrome coronavirus 2
ACE2: Angiotensin-converting enzyme 2
CT-scans: Computer tomography scans
BAL: Bronchoalveolar lavage
EBP: Exhaled breath particles
HPLC–MS/MS: High-performance liquid chromatography-mass spectrometry
PEV: Particles per volume
LFQ: Label free quantification
IGKC: Immunoglobin kappa constant
IGHG1: Immunoglobin heavy constant gamma 1
IGLC2: Immunoglobin lambda constant 3
EPPK1: Epiplakin 1
TF: Serotransferrin
APOA1: Apolipoprotein A-1
CASP14: Caspase-14
CAPN1: Calpain-1
ORM1: Alpha-1-acid glycoprotein 1
SFTPB: Pulmonary surfactant associated protein B
ML: Machine learning
RTLF: Respiratory tract lining fluid
ARDS: Acute respiratory distress syndrome
