# Supplementary figures and images for: Proteomic characteristics and diagnostic potential of exhaled breath particles in patients with COVID-19
Source: Clin Proteomics. 2023 Mar 27;20:13. doi: 10.1186/s12014-023-09403-2 (PMC10040313; doi:10.1186/s12014-023-09403-2)

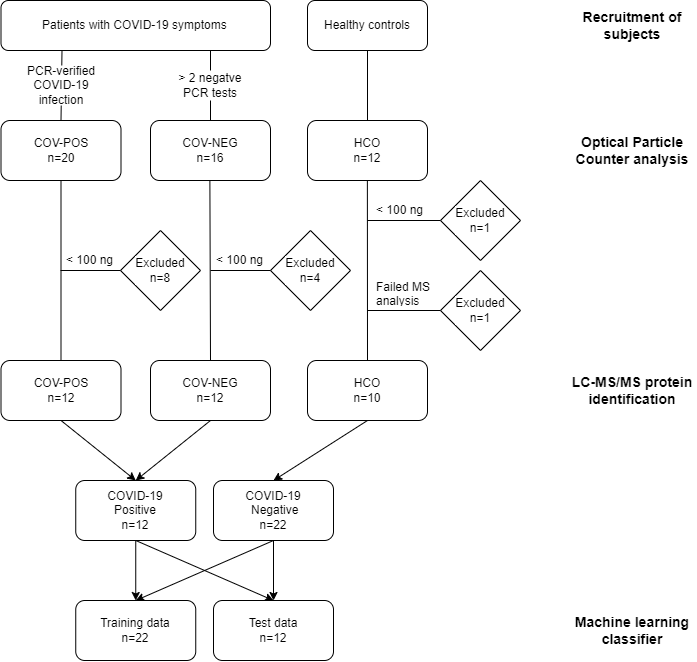

Supplement: Supplementary file 1 — Additional file 1: Figure S1. Flow chart of patient inclusion and sample exclusion. In total 48 subjects were recruited and split into three groups based on symptoms and COVID-19 PCR test results. Subsequently 13 samples were excluded due to insufficient particle collection (< 100 ng of sampled material). One sample in the Healthy control group further failed the mass spectrometry analysis due to technical reasons. The remaining samples where then used for training and testing a machine learning classifier. [file 12014_2023_9403_MOESM1_ESM.png]
