# Supplementary material for: Proteomic characteristics and diagnostic potential of exhaled breath particles in patients with COVID-19
Source: Clin Proteomics. 2023 Mar 27;20:13. doi: 10.1186/s12014-023-09403-2 (PMC10040313; doi:10.1186/s12014-023-09403-2)
Supplement: Supplementary file 2 — Additional file 2: Table S1. LC-MS/MS identified proteins with their statistical differences. Summary of all comparisons between PCR-verified COVID-19 infection (COV-POS), PCR-negative patients with respiratory symptoms (COV-NEG) and healthy controls (HCO) and their adjusted p-value (ANOVA q-value) and Andromeda score from the MaxQuant search engine. [file 12014_2023_9403_MOESM2_ESM.docx]

**Supplementary Table 1 – LC-MS/MS identified proteins with their statistical differences. Summary of all comparisons** between PCR-verified COVID-19 infection (COV-POS), PCR-negative patients with respiratory symptoms (COV-NEG) and healthy controls (HCO) and their adjusted p-value (ANOVA q-value) and Andromeda score from the MaxQuant search engine.

| Gene names | Protein names | ANOVA q-value | Mean difference | | | | Andromeda Score |
| --- | --- | --- | --- | --- | --- | --- | --- |
| A2M | Alpha-2-macroglobulin | 0,194 | 0.0 | | 0.0 | 0.0 | 107 |
| A2ML1 | Alpha-2-macroglobulin-like protein 1 | 0,962 | 0.0 | | 0.0 | 0.0 | 323 |
| ACPP | Prostatic acid phosphatase | 0,920 | 0.0 | | 0.0 | 0.0 | 50 |
| ACTB | Actin, cytoplasmic 1 | 0,157 | 0.0 | | 0.0 | 0.0 | 323 |
| ACTC1 | Actin, alpha cardiac muscle 1 | 0,862 | 0.0 | | 0.0 | 0.0 | 42 |
| ACTG1 | Actin, cytoplasmic 2 | 0,448 | 0.0 | | 0.0 | 0.0 | 54 |
| ACTN4 | Alpha-actinin-4 | 0,046 | 2.0 | | -1.3 | -2.0 | 65 |
| AHNAK | Neuroblast differentiation-associated protein AHNAK | 0,455 | 0.0 | | 0.0 | 0.0 | 37 |
| ALDOA | Fructose-bisphosphate aldolase A | 0,864 | 0.0 | | 0.0 | 0.0 | 49 |
| ALOX12B | Arachidonate 12-lipoxygenase, 12R-type | 0,125 | 0.0 | | 0.0 | 0.0 | 309 |
| AMY2B | Alpha-amylase 2B | 0,685 | 0.0 | | 0.0 | 0.0 | 323 |
| ANXA1 | Annexin A1 | 0,862 | 0.0 | | 0.0 | 0.0 | 323 |
| ANXA2 | Annexin A2 | 0,243 | 0.0 | | 0.0 | 0.0 | 323 |
| APOA1 | Apolipoprotein A-I | 0,036 | 2.4 | | -2.4 | 0.0 | 308 |
| ARG1 | Arginase-1 | 0,044 | -2.1 | | 2.1 | 0.0 | 323 |
| ASAH1 | Acid ceramidase | 0,061 | -1.8 | | 0.0 | 1.8 | 65 |
| ASPRV1 | Retroviral-like aspartic protease 1 | 0,097 | -1.5 | | 1.5 | 0.0 | 292 |
| AZGP1 | Zinc-alpha-2-glycoprotein | 0,541 | 0.0 | | 0.0 | 0.0 | 323 |
| BLMH | Bleomycin hydrolase | 0,071 | -1.7 | | 1.7 | 0.0 | 201 |
| BPIFA1 | BPI fold-containing family A member 1 | 0,135 | 0.0 | | 0.0 | 0.0 | 323 |
| BPIFB1 | BPI fold-containing family B member 1 | 0,558 | 0.0 | | 0.0 | 0.0 | 206 |
| C3 | Complement C3 | 0,202 | 0.0 | | 0.0 | 0.0 | 95 |
| CALML3 | Calmodulin-like protein 3 | 0,291 | 0.0 | | 0.0 | 0.0 | 77 |
| CALML5 | Calmodulin-like protein 5 | 0,961 | 0.0 | | 0.0 | 0.0 | 323 |
| CAPN1 | Calpain-1 catalytic subunit | 0,033 | -2.3 | 2.3 | | 1.4 | 61 |
| CASP14 | Caspase-14 | 0,027 | -2.6 | 2.6 | | 1.5 | 323 |
| CAT | Catalase | 0,036 | -1.4 | 2.2 | | -2.2 | 323 |
| CDSN | Corneodesmosin | 0,308 | 0.0 | 0.0 | | 0.0 | 60 |
| CFL1 | Cofilin-1 | 0,307 | 0.0 | 0.0 | | 0.0 | 99 |
| CLU | Clusterin | 0,810 | 0.0 | 0.0 | | 0.0 | 69 |
| CPA4 | Carboxypeptidase A4 | 0,965 | 0.0 | 0.0 | | 0.0 | 72 |
| CRNN | Cornulin | 0,307 | 0.0 | 0.0 | | 0.0 | 55 |
| CSTA | Cystatin-A | 0,822 | 0.0 | 0.0 | | 0.0 | 323 |
| CTSD | Cathepsin D | 0,995 | 0.0 | 0.0 | | 0.0 | 224 |
| DCD | Dermcidin | 0,369 | 0.0 | 0.0 | | 0.0 | 164 |
| DMBT1 | Deleted in malignant brain tumors 1 protein | 1,000 | 0.0 | 0.0 | | 0.0 | 167 |
| DSC1 | Desmocollin-1 | 0,314 | 0.0 | 0.0 | | 0.0 | 323 |
| DSC3 | Desmocollin-3 | 0,037 | -2.2 | 2.2 | | -1.7 | 270 |
| DSG1 | Desmoglein-1 | 0,163 | 0.0 | 0.0 | | 0.0 | 323 |
| DSP | Desmoplakin | 0,102 | 0.0 | 1.4 | | -1.4 | 323 |
| ECM1 | Extracellular matrix protein 1 | 0,452 | 0.0 | 0.0 | | 0.0 | 116 |
| EEF1A1 | Elongation factor 1-alpha 1 | 0,893 | 0.0 | 0.0 | | 0.0 | 50 |
| EEF2 | Elongation factor 2 | 0,750 | 0.0 | 0.0 | | 0.0 | 85 |
| EIF6 | Eukaryotic translation initiation factor 6 | 0,370 | 0.0 | 0.0 | | 0.0 | 41 |
| ENO1 | Alpha-enolase | 0,997 | 0.0 | 0.0 | | 0.0 | 323 |
| EPPK1 | Epiplakin | 0,029 | 2.6 | -1.4 | | -2.6 | 292 |
| FABP5 | Fatty acid-binding protein,. epidermal | 0,703 | 0.0 | 0.0 | | 0.0 | 219 |
| FGA | Fibrinogen alpha chain | 0,160 | 0.0 | 0.0 | | 0.0 | 126 |
| FGB | Fibrinogen beta chain | 0,062 | 1.4 | -1.4 | | 0.0 | 125 |
| FGG | Fibrinogen gamma chain | 0,097 | 1.4 | -1.4 | | 0.0 | 164 |
| GAPDH | Glyceraldehyde-3-phosphate dehydrogenase | 0,064 | 0.0 | 1.6 | | -1.6 | 323 |
| GGCT | Gamma-glutamylcyclotransferase | 0,134 | 0.0 | 0.0 | | 0.0 | 323 |
| GGH | Gamma-glutamyl hydrolase | 0,100 | -1.4 | 1.4 | | 0.0 | 32 |
| GSDMA | Gasdermin-A | 0,063 | -1.8 | 1.8 | | 0.0 | 215 |
| GSTP1 | Glutathione S-transferase P | 0,822 | 0.0 | 0.0 | | 0.0 | 38 |
| HAL | Histidine ammonia-lyase | 0,281 | 0.0 | 0.0 | | 0.0 | 109 |
| HBA1 | Hemoglobin subunit alpha | 0,961 | 0.0 | 0.0 | | 0.0 | 323 |
| HBB | Hemoglobin subunit beta | 0,812 | 0.0 | 0.0 | | 0.0 | 323 |
| HIST1H2AJ | Histone H2A type 1-J | 0,484 | 0.0 | 0.0 | | 0.0 | 72 |
| HIST1H2BO | Histone H2B type 1-O | 0,066 | 1.5 | -1.5 | | 0.0 | 132 |
| HIST1H4A | Histone H4 | 0,063 | -1.4 | -1.5 | | 1.5 | 15 |
| HP | Haptoglobin | 0,049 | 2.0 | 0.0 | | -2.0 | 188 |
| HPX | Hemopexin | 0,355 | 0.0 | 0.0 | | 0.0 | 84 |
| HSPA1B | Heat shock 70 kDa protein 1B | 0,325 | 0.0 | 0.0 | | 0.0 | 22 |
| HSPA5 | 78 kDa glucose-regulated protein | 0,063 | -1.8 | 0.0 | | 1.8 | 137 |
| HSPB1 | Heat shock protein beta-1 | 1,000 | 0.0 | 0.0 | | 0.0 | 323 |
| IDE | Insulin-degrading enzyme | 0,811 | 0.0 | 0.0 | | 0.0 | 29 |
| IGHA1 | Ig alpha-1 chain C region | 0,022 | 1.3 | -2.9 | | 2.9 | 323 |
| IGHG1 | Ig gamma-1 chain C region | 0,004 | 4.4 | -3.0 | | -4.4 | 323 |
| IGHG2 | Ig gamma-2 chain C region | 1,000 | 0.0 | 0.0 | | 0.0 | 165 |
| IGHG3 | Ig gamma-3 chain C region | 1,000 | 0.0 | 0.0 | | 0.0 | 7 |
| IGJ | Immunoglobulin J chain | 0,267 | 0.0 | 0.0 | | 0.0 | 34 |
| IGKC | Ig kappa chain C region | 0,011 | 3.1 | -3.1 | | 2.2 | 323 |
| IGLC6 | Ig lambda-6 chain C region | 0,034 | 2.5 | -2.5 | | 1.4 | 229 |
| IL36G | Interleukin-36 gamma | 0,361 | 0.0 | 0.0 | | 0.0 | 181 |
| JUP | Junction plakoglobin | 0,061 | -1.7 | 1.7 | | 0.0 | 323 |
| KLK7 | Kallikrein-7 | 0,122 | 0.0 | 0.0 | | 0.0 | 20 |
| KPRP | Keratinocyte proline-rich protein | 0,063 | -1.6 | 1.6 | | 0.0 | 323 |
| LCN1 | Lipocalin-1 | 0,160 | 0.0 | 0.0 | | 0.0 | 65 |
| LDHA | L-lactate dehydrogenase A chain | 0,225 | 0.0 | 0.0 | | 0.0 | 64 |
| LGALS7 | Galectin-7 | 0,818 | 0.0 | 0.0 | | 0.0 | 107 |
| LMNA | Prelamin-A/C | 0,992 | 0.0 | 0.0 | | 0.0 | 125 |
| LTF | Lactotransferrin | 0,072 | 0.0 | -1.7 | | 1.7 | 323 |
| LYZ | Lysozyme C | 0,067 | -1.7 | 0.0 | | 1.7 | 103 |
| MUC5B | Mucin-5B | 0,277 | 0.0 | 0.0 | | 0.0 | 70 |
| MYH9 | Myosin-9 | 0,617 | 0.0 | 0.0 | | 0.0 | 323 |
| NCCRP1 | F-box only protein 50 | 0,813 | 0.0 | 0.0 | | 0.0 | 41 |
| ORM1 | Alpha-1-acid glycoprotein 1 | 0,012 | 2.8 | -2.8 | | -2.6 | 165 |
| PEBP1 | Phosphatidylethanolamine-binding protein 1 | 1,000 | 0.0 | 0.0 | | 0.0 | 39 |
| PIGR | Polymeric immunoglobulin receptor | 0,048 | -1.6 | -1.6 | | 1.6 | 109 |
| PIP | Prolactin-inducible protein | 0,610 | 0.0 | 0.0 | | 0.0 | 219 |
| PKM | Pyruvate kinase PKM | 0,991 | 0.0 | 0.0 | | 0.0 | 94 |
| PKP1 | Plakophilin-1 | 0,041 | -2.1 | 2.1 | | 0.0 | 323 |
| PLBD1 | Phospholipase B-like 1 | 0,049 | -1.9 | 1.9 | | 0.0 | 76 |
| PNP | Purine nucleoside phosphorylase | 0,588 | 0.0 | 0.0 | | 0.0 | 159 |
| POF1B | Protein POF1B | 0,153 | 0.0 | 0.0 | | 0.0 | 184 |
| PRDX1 | Peroxiredoxin-1 | 0,792 | 0.0 | 0.0 | | 0.0 | 174 |
| PRDX2 | Peroxiredoxin-2 | 0,746 | 0.0 | 0.0 | | 0.0 | 113 |
| PRH1 | Salivary acidic proline-rich phosphoprotein 1/2 | 0,359 | 0.0 | 0.0 | | 0.0 | 133 |
| PSMA2 | Proteasome subunit alpha type-2 | 0,269 | 0.0 | 0.0 | | 0.0 | 109 |
| PSMA3 | Proteasome subunit alpha type-3 | 0,043 | -2.1 | 2.1 | | 1.7 | 88 |
| PSMA5 | Proteasome subunit alpha type-5 | 0,062 | -1.6 | 1.6 | | 1.3 | 53 |
| PSMA6 | Proteasome subunit alpha type-6 | 0,717 | 0.0 | 0.0 | | 0.0 | 32 |
| PSMA7 | Proteasome subunit alpha type-7 | 0,118 | 0.0 | -1.3 | | 1.3 | 64 |
| PSMB1 | Proteasome subunit beta type-1 | 0,925 | 0.0 | 0.0 | | 0.0 | 14 |
| PSMB3 | Proteasome subunit beta type-3 | 0,997 | 0.0 | 0.0 | | 0.0 | 82 |
| PSMB5 | Proteasome subunit beta type-5 | 0,101 | -1.4 | 0.0 | | 1.4 | 36 |
| RNASE7 | Ribonuclease 7 | 0,814 | 0.0 | 0.0 | | 0.0 | 31 |
| S100A14 | Protein S100-A14 | 0,047 | -1.9 | 1.9 | | 0.0 | 227 |
| S100A16 | Protein S100-A16 | 0,384 | 0.0 | 0.0 | | 0.0 | 10 |
| S100A7 | Protein S100-A7 | 0,780 | 0.0 | 0.0 | | 0.0 | 323 |
| S100A8 | Protein S100-A8 | 0,722 | 0.0 | 0.0 | | 0.0 | 298 |
| S100A9 | Protein S100-A9 | 0,851 | 0.0 | 0.0 | | 0.0 | 323 |
| SBSN | Suprabasin | 0,897 | 0.0 | 0.0 | | 0.0 | 323 |
| SCGB2A1 | Mammaglobin-B | 0,955 | 0.0 | 0.0 | | 0.0 | 31 |
| SDR9C7 | Short-chain dehydrogenase/reductase family 9C member 7 | 0,848 | 0.0 | 0.0 | | 0.0 | 11 |
| SERPINA1 | Alpha-1-antitrypsin | 0,044 | 2.2 | -2.2 | | 0.0 | 323 |
| SERPINA12 | Serpin A12 | 0,096 | -1.5 | 1.5 | | 0.0 | 173 |
| SERPINA3 | Alpha-1-antichymotrypsin | 0,625 | 0.0 | 0.0 | | 0.0 | 49 |
| SERPINB12 | Serpin B12 | 0,310 | 0.0 | 0.0 | | 0.0 | 323 |
| SERPINB13 | Serpin B13 | 0,143 | 0.0 | 0.0 | | 0.0 | 115 |
| SERPINB2 | Plasminogen activator inhibitor 2 | 0,875 | 0.0 | 0.0 | | 0.0 | 75 |
| SERPINB3 | Serpin B3 | 0,200 | 0.0 | 0.0 | | 0.0 | 323 |
| SERPINB4 | Serpin B4 | 0,560 | 0.0 | 0.0 | | 0.0 | 281 |
| SERPINB7 | Serpin B7 | 0,312 | 0.0 | 0.0 | | 0.0 | 40 |
| SFTPA1 | Pulmonary surfactant-associated protein A1 | 0,145 | 0.0 | 0.0 | | 0.0 | 323 |
| SFTPB | Pulmonary surfactant-associated protein B | 0,016 | -2.7 | -2.2 | | 2.7 | 69 |
| TAGAP | T-cell activation Rho GTPase-activating protein | 0,436 | 0.0 | 0.0 | | 0.0 | 2 |
| TF | Serotransferrin | 0,021 | 2.9 | -2.9 | | 0.0 | 323 |
| TGM1 | Protein-glutamine gamma-glutamyltransferase K | 0,043 | -1.8 | 1.8 | | -1.5 | 261 |
| TGM3 | Protein-glutamine gamma-glutamyltransferase E | 0,821 | 0.0 | 0.0 | | 0.0 | 323 |
| TGM5 | Protein-glutamine gamma-glutamyltransferase 5 | 0,864 | 0.0 | 0.0 | | 0.0 | 310 |
| TKT | Transketolase | 0,140 | 0.0 | 0.0 | | 0.0 | 142 |
| TOLLIP | Toll-interacting protein | 1,000 | 0.0 | 0.0 | | 0.0 | 11 |
| TPI1 | Triosephosphate isomerase | 0,239 | 0.0 | 0.0 | | 0.0 | 36 |
| TPM3 | Tropomyosin alpha-3 chain | 0,998 | 0.0 | 0.0 | | 0.0 | 39 |
| TPP1 | Tripeptidyl-peptidase 1 | 0,996 | 0.0 | 0.0 | | 0.0 | 102 |
| TTR | Transthyretin | 0,291 | 0.0 | 0.0 | | 0.0 | 245 |
| TUBA1C | Tubulin alpha-1C chain | 0,856 | 0.0 | 0.0 | | 0.0 | 62 |
| TUBB2B | Tubulin beta-2B chain | 1,000 | 0.0 | 0.0 | | 0.0 | 105 |
| TXN | Thioredoxin | 0,048 | -1.3 | -1.8 | | 1.8 | 84 |
| UBA52 | Ubiquitin-60S ribosomal protein L40 | 0,065 | -1.8 | 1.8 | | 0.0 | 323 |
| VCL | Vinculin | 0,040 | -1.8 | -1.8 | | 1.8 | 52 |
| YOD1 | Ubiquitin thioesterase OTU1 | 0,448 | 0.0 | 0.0 | | 0.0 | 35 |
| YWHAZ | 14-3-3 protein zeta/delta | 0,956 | 0.0 | 0.0 | | 0.0 | 141 |
| ZG16B | Zymogen granule protein 16 homolog B | 0,043 | 0.0 | -2.2 | | 2.2 | 323 |
